# Susceptibility to Metabolic Diseases in COVID-19: To be or Not to be an Issue

**DOI:** 10.3389/fmolb.2022.803314

**Published:** 2022-02-03

**Authors:** Maryam Kaviani, Somayeh Keshtkar, Saeede Soleimanian, Fatemeh Sabet Sarvestani, Negar Azarpira, Sara Pakbaz

**Affiliations:** ^1^ Transplant Research Center, Shiraz University of Medical Sciences, Shiraz, Iran; ^2^ Molecular Dermatology Research Center, Shiraz University of Medical Sciences, Shiraz, Iran; ^3^ Department of Laboratory Medicine and Pathobiology, Faculty of Medicine, University of Toronto, Toronto, ON, Canada

**Keywords:** COVID-19, recovered, susceptibility, metabolic diseases, molecular mechanisms

## Abstract

Despite the passage of more than 17 months from the beginning of the COVID-19 pandemic, challenges regarding the disease and its related complications still continue in recovered patients. Thus, various studies are underway to assay the long-term effects of COVID-19. Some patients, especially those with severe symptoms, experience susceptibility to a range of diseases and substantial organ dysfunction after recovery. Although COVID-19 primarily affects the lungs, multiple reports exist on the effect of this infection on the kidneys, cardiovascular system, and gastrointestinal tract. Studies have also indicated the increased risk of severe COVID-19 in patients with diabetes. On the other hand, COVID-19 may predispose patients to diabetes, as the most common metabolic disease. Recent studies have shown that Severe Acute Respiratory Syndrome Coronavirus-2 (SARS-CoV-2) binds to Angiotensin-Converting Enzyme 2 (ACE2) receptors, which are expressed in the tissues and organs involved in regulating the metabolic status including pancreas, adipose tissue, gastrointestinal tract, and kidneys. Therefore, SARS-CoV-2 may result in metabolic disturbance. However, there are still many unknowns about SARS-CoV-2, which are required to be explored in basic studies. In this context, special attention to molecular pathways is warranted for understanding the pathogenesis of the disease and achieving therapeutic opportunities. Hence, the present review aims to focus on the molecular mechanisms associated with the susceptibility to metabolic diseases amongst patients recovered from COVID-19.

## Introduction

Investigation of the history of medicine indicates that the outbreak of viral respiratory infections is not a new phenomenon. During the past century, there have been several reports on the prevalence of respiratory infections including Severe Acute Respiratory Syndrome Coronavirus-1 (SARS-CoV-1) (Tratner 2003) and Middle East Respiratory Syndrome Coronavirus (MERS-CoV) (Lancet 2013). Since December 2019, the Coronavirus Disease-19 (COVID-19) pandemic has become the most challenging issue throughout the world. Many studies have been done on the nature of Severe Acute Respiratory Syndrome Coronavirus-2 (SARS-CoV-2) and its therapeutic strategies (Cardamone and Donatiello 2020; Schlosser et al., 2020).

Although COVID-19 has been primarily recognized as a respiratory infection, clinical evidence has demonstrated that SARS-CoV-2 affects multiple organs (de-Madaria et al., 2020; Ridruejo and Soza 2020; Sharma et al., 2020; Hunt et al., 2021; Nalbandian et al., 2021). Therefore, post- COVID-19 complications are predictable in recovered patients. For instance, it has been hypothesized that COVID-19 may result in metabolic syndrome. Metabolic syndrome refers to a cluster of conditions that rises the risk of cardiovascular disorders and type II diabetes (Eckel et al., 2005). The International Diabetes Federation (IDF) reported the metabolic syndrome criteria in 2006 (Zafar et al., 2018), which have been summarized in Table 1.

**TABLE 1:** Diagnostic criteria for metabolic syndrome.

Obesity	Central obesity as defined by ethnicity/race-specific waist circumference, but can be assumed if Body Mass Index (BMI) ≥ 30 kg/m^2^ and two or more of the following
Fasting plasma glucose	≥100 mg/dl or being on treatment for diabetes mellitus
Blood pressure	≥130/85 mmHg or being on anti-hypertensive drugs
Triglycerides	≥150 mg/dl or being on lipid lowering agents
High density lipoprotein-cholesterol	<40 mg/dl for males, < 50 mg/dl for females, or being on treatment for dyslipidemia

Recent studies have indicated that SARS-CoV-2 binds to Angiotensin-Converting Enzyme 2 (ACE2) receptors. These receptors mediate the transmission and infection processes of the virus. The virus has spike (S) glycoprotein on its surface, which can attach to the cell surface in order to turn this glycoprotein into two domains (S1 and S2) and exert its effect. This separation is allowed by FURIN and Transmembrane Serine Protease (TMPRSS2). In this way, S1 binds to ACE2 receptors and S2 binds to the cell membrane, allowing the virus to enter the cell through endocytosis (Ding and Liang 2020; Mönkemüller et al., 2020). In fact, after the virus binds to ACE2, the receptor’s ectodomain is cleaved. Then, the transmembrane domain is internalized by endocytosis. On the other hand, the whole ACE2 molecule can enter the cell with the virus (Li et al., 2005). ACE2 is expressed in the tissues and organs involved in regulating the metabolic status including pancreas (Liu et al., 2020), gastrointestinal tract (Qi et al., 2020; Zhang et al., 2020), liver (Chai et al., 2020), and kidneys (Qi et al., 2020). Thus, metabolic syndrome may be a potential complication in patients recovered from SARS-CoV-2 (Lee et al., 2021; Sathish et al., 2021).

Zhang et al. reported abnormal plasma metabolomic profiles in patients recovered from COVID-19 without any underlying diseases. They found that some clinical indicators were abnormal in patients recovered from moderate and severe COVID-19 3 months after discharge. These parameters included taurine, succinic acid, hippuric acid, some indoles, and lipid species that were associated with the functions of kidneys, lungs, heart, liver, and the coagulation system (Zhang et al., 2021). Moreover, Holmes et al. referred to the post-acute COVID-19 syndrome in non-hospitalized patients with COVID-19 in the post-acute phase of the disease. They analyzed the blood samples of 27 patients 3 month after acute COVID-19 infection. The findings indicated disruptions in several parameters including taurine and glutamine/glutamate ratio. These results demonstrated the possibility of damages to the liver and muscles in non-hospitalized patients with COVID-19 in the post-acute phase (Holmes et al., 2021). On the other hand, the results of a systematic review and meta-analysis revealed that severe COVID-19 was associated with elevated blood glucose levels and a slight increase in HbA1c levels (Chen Juan et al., 2020). Although limited studies have been conducted on patients’ follow-up after COVID-19 infection, the results obtained so far have shown the fluctuation of multiple metabolic indicators in patients recovered from COVID-19, especially its severe form.

**FIGURE 1:**
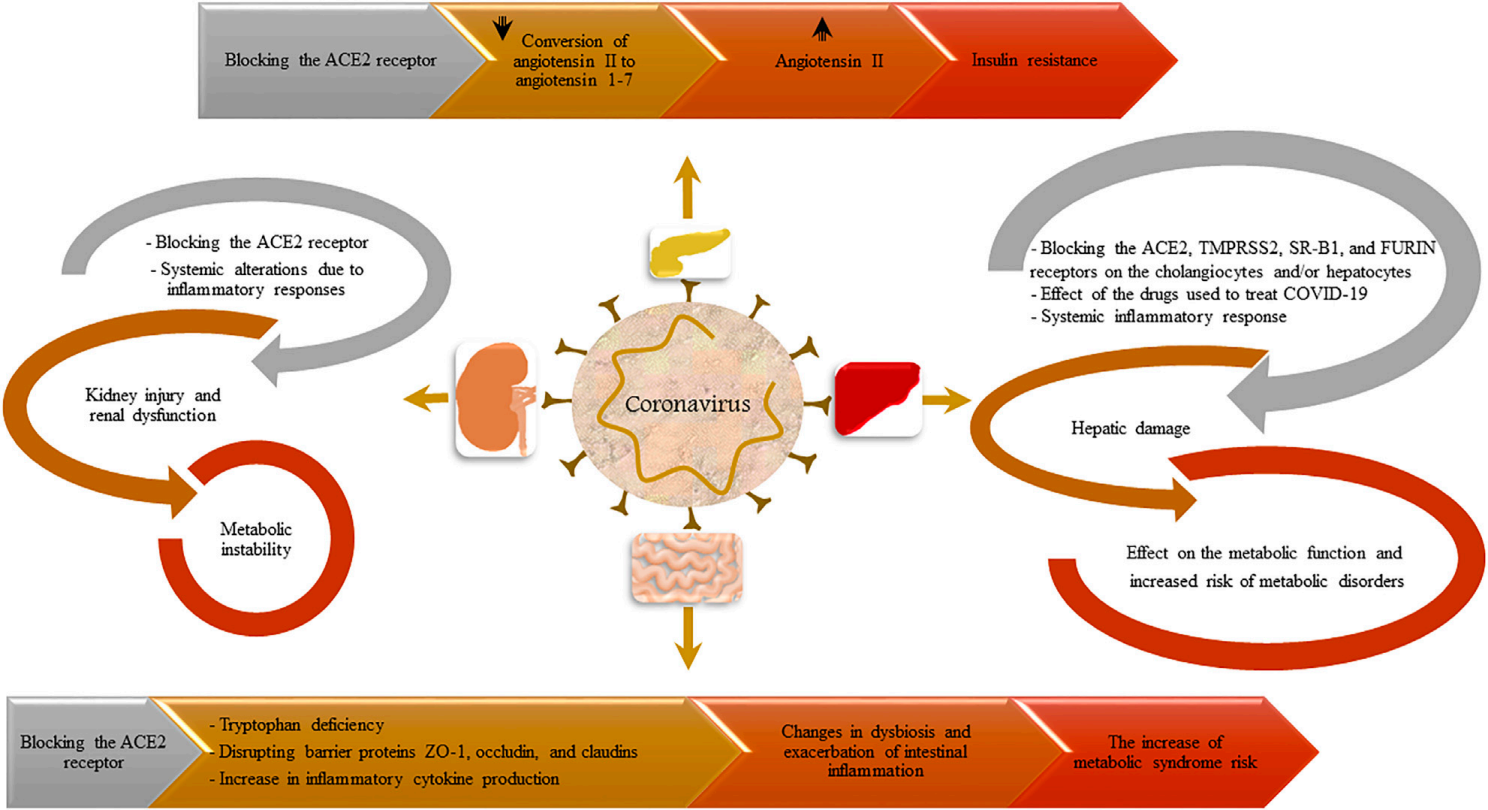
A summary of the possible effects of COVID-19 infection on the development of metabolic syndrome.

While a variety of studies have shown that several underlying diseases including diabetes (Gupta et al., 2020), kidney diseases (Rabb 2020), and cardiovascular disorders (Li Jialing et al., 2020) increase the severity and mortality in SARS-CoV-2 infection, the association between COVID-19 and the onset of some diseases needs to be determined. Hence, the present review aims to investigate the mechanisms involved in the susceptibility of recovered patients to metabolic syndrome. In this regard, the organs involved in metabolic syndrome including pancreas, gastrointestinal tract, kidneys, and liver (Figure 1) have been discussed and the relationships between these organs and COVID-19 have been addressed.

### The Effect of COVID-19 on the Pancreas

There is a growing body of evidence on the bidirectional relationship between diabetes and COVID-19. This implies that while patients with diabetes experience more severe forms of COVID-19, patients with COVID-19 may develop metabolic complications and diabetes (Rubino et al., 2020).

Diabetes has been introduced as a risk factor for higher morbidity and mortality in patients with COVID-19. Evidence has revealed that COVID-19 may affect the pathophysiology of diabetes through increased ACE-2 expression, increased paired basic amino acid cleaving enzyme (FURIN), impaired T-cell function, and increased interleukin-6 (Singh et al., 2020). Therefore, patients with diabetes need special clinical management to avoid the progressive damage. On the other hand, due to the diabetogenic nature of COVID-19, evaluation of recovered patients is essential. In this regard, investigation of the underlying mechanisms that lead to the onset of diabetes in patients with COVID-19 can be beneficial for the prevention and management of the issue.

It seems that the most important mechanism related to the COVID-19 complications is the expression pattern of the Receptor-Binding Domain (RBD) of ACE2 on cells, as a binding site for SARS-COV-2 (El-Huneidi et al., 2021). Studies have shown that ACE2 is expressed in the β-cells of the pancreas. Thus, SARS-COV-2 may exert direct effects on pancreatic β-cells, subsequently resulting in glucose and metabolic instability. Analysis of the GTEx database revealed a higher expression of ACE2 in the pancreas than in lungs. Moreover, evaluation of ACE2 distribution in the pancreas showed the expression of this receptor in both exocrine and endocrine parts (Liu et al., 2020). Liu et al. evaluated pancreatic injury after COVID-19 infection in a cohort study on 121 patients. They found that pancreatic injury occurred mainly in severe COVID-19 infection, which was suggested to be associated with ACE2 expression in the pancreas (Liu et al., 2020). Researchers also focused on the potential of COVID-19 to affect the endocrine pancreas through the activation of NHA2 as a Na+/H+ Exchanger (NHE) (Cure E. and Cumhur Cure M., 2020). Based on the previous studies, NHA2 is present in human pancreatic β-cells. Additionally, *in vitro* and *in vivo* assays have indicated that NHA2 is critical for insulin secretion in β-cells (Deisl et al., 2013). Accordingly, NHA2 deficiency is accompanied by a decrease in insulin secretion. On the other hand, increased NHA2 activity leads to insulin resistance. In COVID-19 infection, conversion of angiotensin II to angiotensin 1–7 decreases after blocking the ACE2 receptors by the virus. The elevated level of angiotensin II leads to the increase of insulin resistance. Cure et al. also suggested that the chymase pathway was probably involved in the formation of angiotensin II. Chymase is a major non-ACE pathway in the generation of angiotensin II. Through this alternative pathway, angiotensin-(1–12) and angiotensin I are converted to angiotensin II by the proteolytic enzymatic activity of chymase. Although the role of this pathway in COVID-19 disease is not well known, chymase may be a potential mediator. Garvin et al. proposed the positive interplay between the kallikrein-bradykinin system and chymase in COVID-19 patients (Garvin et al., 2020).

Activation of NHE by angiotensin II results in the increase of Na+ and H+ exchange as well as Na+ and Ca2+ exchange. In this regard, H+ ions accumulate out of the cell and make the microenvironment acidic and hypoxic. Subsequent to hypoxia, reactive oxygen radicals increase. Oxidative stress plays a fundamental role in metabolic syndrome. Besides, accumulation of reactive products can develop insulin resistance. Increased levels of Ca2+ and Na+ also damage cells (Cure E. and Cumhur Cure M., 2020).

Hypoxia is yet another factor that can affect the function of the endocrine pancreas. In hypoxia due to COVID-19 infection, the lactate level elevates. The increased level of lactate causes the increment of H^+^ ions, which move inside cells and make NHE overactive (Cure Erkan and Cumhur Cure Medine, 2020). Moreover, there is a dynamic relationship between lactate and glucose. Lactate has been introduced as the major gluconeogenic precursor that increases glucose production (Bergman et al., 2000; Emhoff et al., 2013). This situation can impose an additional burden on the pancreas and can be destructive in severe and prolonged COVID-19 infection.

In conclusion, the new onset of diabetes in non-diabetic patients and exacerbation of diabetes in those suffering from the disease have been reported subsequent to COVID-19 infection. According to the abovementioned information, paying attention to the basic mechanisms involved in the interaction between COVID-19 infection and diabetes is a useful guide for taking preventive and therapeutic measures. Considering the high expression of ACE2 receptors in pancreatic β-cells, they may be damaged by SARS-CoV-2. Thus, early diagnosis and careful management of patients with COVID-19 are needed to avoid further complications related to hyperglycemia and metabolic imbalance. Moreover, targeting ACE2 has been proposed to mitigate infectivity (Jia et al., 2021). Recently, strategies related to ACE2 targeting such as decreasing Ang II to decoy receptor (Haschke et al., 2013), blocking ACE2 (Huentelman et al., 2004), enhancing ACE2 shedding (Jia et al., 2009), and facilitating ACE2 internalization (Touret et al., 2020) have been used for the prevention and treatment of COVID-19. All in all, the diabetogenic potential of COVID-19 requires further studies to determine its main characteristics and persistence, especially in cases with new onset diabetes.

### The Effect of COVID-19 on the Gastrointestinal Tract

The primary function of the gut is to supply the body with energy via building elements and water/electrolytes. Energy homoeostasis consists of two principal functional states; i.e., catabolic state and anabolic state. The switch between these two metabolic conditions is related to meal ingestion and digestion/absorption and is thus dependent on the functional state of the gastrointestinal tract (Fändriks 2017). The gut microbiota plays a pivotal role in maintaining the homeostasis of human health (Festi et al., 2014). The human intestinal microbiota composition is the result of a bilateral interaction between the host and its microbial consortium. In fact, the composition and stability of the intestinal microbiota are determined by nutrition, drugs, diseases, etc. (Hur and Lee 2015). Dysbiosis of the gut microbiota caused by various factors leads to extensive physiological changes and increases the risk of metabolic syndrome (Festi et al., 2014).

It mainly infects lung cells, but may involve the gastrointestinal tract that has ACE2 receptors in the epithelial cells of the intestinal mucosa, but not in the esophagus and stomach (He et al., 2006; Gu and Korteweg 2007; Xiao et al., 2020). In the gastrointestinal tract, ACE2 has been described as a key regulator of dietary amino acid homeostasis, expression of antimicrobial peptides, local innate immunity, and gut microbial ecology (Hashimoto et al., 2012). In fact, ACE2 is associated with the amino acid carrier, B0AT1, which regulates the intestinal flora. This occurs because this transporter allows the absorption of tryptophan, which stimulates the mTOR path to produce antimicrobial peptides. Thus, the SARS-CoV-2 infection changes and blocks the ACE2 receptors in the brush edge, causing tryptophan deficiency and lower production of antimicrobial peptides, which can in turn cause changes in the intestinal microbiota and result in inflammation (Ding and Liang 2020; Mönkemüller et al., 2020). On the other hand, the interaction between SARS-CoV-2 and ACE2 in the gastrointestinal tract may lead to damage to the barrier function *via* disrupting barrier proteins ZO-1, occludin, and claudins as well as an increase in inflammatory cytokines production, which may in turn lead to dysbiosis and exacerbation of intestinal inflammation (Fernández-Blanco et al., 2015; Chen Li et al., 2020). Besides, intestinal inflammation may augment dysbiosis and damage the intestinal mucosal barrier function. Moreover, intestinal lymphocytes, dendritic cells, and macrophages may perpetuate the cytokine storm (Fernández-Blanco et al., 2015).

Preventing interaction between the SARS-CoV-2 RBD and ACE2 may be an effective strategy to prevent viral infections. In this way, ACE2 inhibitors can affect intestinal amino acid metabolism, antibacterial peptide secretion, intestinal microbial homeostasis, and innate immunity through the mTOR pathway (Hashimoto et al., 2012; Li et al., 2020). Furthermore, metabolic syndrome can be improved through the alteration of intestinal microbiota via the administration of probiotics and prebiotics that increase the short chain fatty acid production as well as by using a diet with 30% fat content and a high fruit and vegetable content such as the Mediterranean diet (Santos-Marcos et al., 2019).

### The Effect of COVID-19 on Kidneys

Evidence has revealed an association between Chronic Kidney disease (CKD) and the metabolic syndrome (Wu et al., 2021). A significant correlation has also been reported between kidney insufficiency and diabetes, obesity, and hypertension (Culleton et al., 1999). The metabolic syndrome related to renal dysfunction results from various factors (Peralta et al., 2006) including hyperinsulinemia (insulin resistance), activation of the renin-angiotensin system, abnormal production of growth factors, inflammation, and oxidative stress. Among these factors, hyperinsulinemia is the most important risk factor for the metabolic syndrome associated with CKD, which induces renal damage mechanisms (El-Achkar et al., 2005). On the other hand, kidney is a major organ in the regulation of glucose and plays a key role in glucose reabsorption and filtration (Rabkin et al., 1984). Furthermore, kidneys primarily metabolize insulin, resulting in the degradation of circulating insulin (Mather and Pollock 2011). Considering renal function in the clearance of insulin, renal insufficiency affects glucose homeostasis and alters insulin metabolism (Duckworth et al., 1998; Garla et al., 2017). According to these findings, CKD and diabetes are complicatedly intertwined. This implies that the management of metabolic abnormalities due to CKD is a major challenge for clinicians (Garla et al., 2017). Therefore, any reason for renal complication may lead to the disturbance of glucose metabolism and, subsequently, the development of metabolic syndrome.

Renal injury results from viral infections through direct and indirect invasion by offending viruses, leading to cytopathic injury followed by renal cells injury (Prasad and Patel 2018). Re-emergence of different viral infections has been a recent pattern associated with kidney diseases (Morens et al., 2004). In this context, the consequences of the COVID-19 pandemic have been overrunning, being a challenge facing healthcare systems worldwide (Cheng et al., 2020). Acute Kidney Injury (AKI), as a major complication of COVID-19, arises from multifactorial mechanisms and increases the risk of mortality in these patients (Lili et al., 2020; Gupta et al., 2021). Furthermore, CKD develops in AKI-recovered patients as a long-term renal dysfunction. Notably, kidney transplant patients as well as individuals with CKD are at an increased risk of severe COVID-19 (Jager et al., 2020), and patients with severe COVID-19 are at a high risk of AKI as a frequent complication (Ng et al., 2021). It has been revealed that podocytes and proximal tubular epithelial cells are present as a kidney tropism upon SARS-CoV-2 infection. Progression of the renal injury initiates through changes in the metabolism of kidney cells, resulting in metabolic diseases (Yan et al., 2018; Legouis et al., 2020). Generally, two scenarios can be considered for kidney injury and decreased function of this organ in patients with COVID-19. The main scenario is the direct infection of renal cells with SARS-CoV-2, which is in accordance with the fact that kidney is one of the particular targets of COVID-19 through ACE2 receptors. In the second scenario, systemic alterations in metabolism stemming from the immune system response may lead to AKI in patients with COVID-19 (Andrade Silva et al., 2021a). Based on a follow-up study on COVID-19-recovered patients, the kidney expresses the ACE2 receptors like the heart, lung, and gut (Diao et al., 2020). Moreover, the Proximal Convoluted Tubule (PCT) of the host renal cells consists of two important receptors, namely ACE2 and Transmembrane protease Serine 2 (TMPRSSs), for viral entry (Pan et al., 2020). After entry, accumulation of the extracellular matrix occurs and causes diuresis and proliferation of kidney cells, finally leading to AKI (Balachandar et al., 2020). Intriguingly, a recent investigation found that AKI following COVID-19 was associated with the increased binding of the virus to the ACE2 receptors (Perico et al., 2020). Moreover, the SARS-CoV-2 entry process was promoted through the proteolytic activity of TMPRSS (Shen et al., 2017). After SARS-CoV-2 enters renal cells, in case the cells’ inflammatory system can pass the threshold for activating the Nucleotide-binding Domain (NOD)-like Receptor Protein 3 (NLRP3) inflammasome, cell death will occur via pyroptosis (Qing et al., 2020). The NLRP3 inflammasome is a cytosolic protein complex, which is a crucial regulator of the inflammation pathway and the innate immune response (Martinon et al., 2009). Hence, inflammasome has a central role in the pathogenesis of several inflammatory disorders such as diabetes, atherosclerosis, and arthritis (Schroder et al., 2010; Davis et al., 2011). There are three pathways for this intracellular protein complex to become activated. In the first pathway, the spike protein SARS-CoV-2 directly stimulates the NLRP3 inflammasome by binding to renal cells via the ACE2 receptors. In the second pathway, activation of the Renin–Angiotensin–Aldosterone System (RAAS) increases the level of angiotensin II, which induces the NLRP3 inflammasome after attaching to the angiotensin I receptor. In the third pathway, some cleavage fragments including C3a and C5a as anaphylatoxins and C5bC9 as a membrane attack complex are released through the direct interaction between the Complement Cascade (ComC) and SARS-CoV-2, which may lead to the activation of the NLRP3 inflammasome (Ratajczak and Kucia 2020). It should be noted that the hyper-activation of the NLRP3 inflammasome is a significant problem for human host cells, resulting in the perturbation of mitochondrial function, cell death, and severe kidney tissue injury (van den Berg and Te Velde 2020). This results from the fact that NLRP3 activation leads to caspase-1 cleavage, induction of other Damage-Associated Molecular Patterns (DAMPs), and production of pro-inflammatory cytokines IL-1β and IL-18 (Freeman and Swartz 2020). Therefore, this activation should be downregulated through increasing antibodies and adequate adaptive responses (Ros et al., 2020). The overstimulation of the NLRP3 inflammasome and cell death pathways can influence renal functionality, leading to inflammation and kidney damage, triggering alterations in tubular epithelial cell metabolism, and finally resulting in metabolic disturbance (Li et al., 2019). Yet, further studies are required to clarify the molecular mechanisms involved in metabolic dysfunction during COVID-19 progression.

Toll-Like Receptors (TLRs) are a type of pattern recognition receptors, which have been revealed as another potential receptor binding to the S protein of SARS-CoV-2 (Choudhury and Mukherjee 2020). Based on the recent studies, the interaction between TLR-4 receptors and SARS-CoV-2 results in the induction of inflammatory responses and profoundly affects the metabolism of mitochondrial, lipid, and glycolytic homeostasis in antigen presenting cells including macrophages and dendritic cells, eventually affecting the systemic metabolism (Perrin-Cocon et al., 2018; Lauterbach et al., 2019). Furthermore, activation of TLR4 leads to a severe inflammation pathway in kidneys, thereby causing AKI (Andrade-Silva et al., 2018; Andrade Silva et al., 2021b). However, it remains unknown whether metabolic dysfunction after recovery from COVID-19 can be correlated to TLRs signaling in kidneys. Therefore, recovered patients are recommended to be followed up to ensure the absence of underlying conditions stemming from COVID-19. In this context, creatinine levels have to be monitored frequently for acute kidney damage after recovery from COVID-19. Now, the key question rises whether such compounds as NLRP3 inflammasome inhibitors and ComC inhibitors that mitigate the innate immunity activation can be considered a therapeutic option. In this regard, mesenchymal stromal cells can be nominated as the potential modulators of the immune system.

### The Effect of COVID-19 on the Liver

Liver is known as a key organ in the regulation of necessary routs for the maintenance of systemic metabolic hemostasis. The regulation of lipid and glucose hemostasis is orchestrated by hepatocytes. Following metabolic imbalance between glucose and lipid metabolism, hepatic steatosis occurs. In fact, whenever the amount of liver fat reaches more than 5% of the liver weight, hepatic steatosis occurs, irrespective of alcohol consumption (Rosselli et al., 2014). Accordingly, Nonalcoholic Fatty Liver Disease (NAFLD) has been identified as the hepatic manifestation of metabolic syndrome, because it is closely related to the impaired metabolism of fatty acids, lipoproteins, and glucose (Rosselli et al., 2014; Godoy-Matos et al., 2020). NAFLD is in fact the most common liver disease that is associated with metabolic and cardiovascular disorders including insulin resistance, hypertension, dyslipidemia, and type II diabetes. Recently, a number of international experts have suggested definition criteria for the metabolic dysfunction-associated fatty liver disease (MAFLD). The definition of MAFLD includes hepatic steatosis confirmed through histological, imaging, or blood biomarkers together with at least one of the three main criteria of metabolic disorder; i.e., overweight/obesity, type II diabetes, and the presence of metabolic dysregulation. According to epidemiological assessments, the highest prevalence of MAFLD has been detected in Middle East and South America where almost half of the population have the metabolic syndrome. It is noteworthy that advanced hepatic fibrosis is observed in about 7% of people with hepatic steatosis and its incidence is almost doubled in the presence of metabolic syndrome (Godoy-Matos et al., 2020).

The prevalence of liver injury following COVID-19 has been reported to be 21.5–46%. Additionally, it has been found to be associated with multifactorial and heterogeneous reasons that are required to be monitored closely and continuously. Thus, in the context of COVID-19, it should be explored whether the liver damage is related to the side effects of the drugs used for COVID-19 treatment, the direct effect of the virus, an underlying liver disease, and/or combination in the disease course. Liver damage has been supposed to be more severe in patients with NAFLD. Up to now, different mechanism of liver injury caused by COVID-19 infection have been proposed (Yu et al., 2021). The first mechanism may be a direct damage to the liver via ACE2 receptors. ACE2 is widely expressed in hepatobiliary system cells including hepatocytes and cholangiocytes (Alqahtani and Schattenberg 2020). Two other receptors called TMPRSS2 and FURIN are also important for infection in hepatic cells (Marjot et al., 2021). The expression of ACE2 is higher in cholangiocytes than in hepatocytes. TMPRSS2 and FURIN are expressed in many liver cells types. However, very few hepatocytes express both ACE2 and TMPRSS2. Cholangiocytes play a key role in liver regeneration and immune response, and their dysfunction can lead to hepatic damage. In a recent study, Chai et al. evaluated the expression of ACE2 in healthy human liver tissues and demonstrated that viruses might directly bind to ACE2-positive cholangiocytes, but not to hepatocytes. Thus, they suggested that the liver damage in patients with SARS and COVID-19 might not be associated with the direct infection of hepatocytes. Cholangiocyte dysfunction by viral infection as well as by other causes like the drugs used for treatment or the systemic inflammatory response induced by pneumonias could lead to liver injury. Alkaline Phosphatase (ALP) and Gamma Glutamyltranspeptidase (GGT), as cholangiocytes injury markers, increased in some patients with COVID-19, supporting the role of cholangiocytes dysfunction in liver injury in these patients (Alqahtani and Schattenberg 2020; Marjot et al., 2021). Recently, Zhao et al. created a human liver ductal hepatocyte organoid model that was permissible to SARS-CoV-2 infection and replication. They observed that SARS-CoV-2-infected cholangiocytes directly impaired their barriers and bile acid transporting functions that resulted in hepatobiliary injury, confirming the strong role of cholangiocytes in the liver injury induced by SRAS-CoV-2 (Marjot et al., 2021).

Apart from the specific role of ACE-2 receptors, additional receptors have been found to play roles in virus entry. One of these receptors is the high-density lipoprotein Scavenger Receptor B-type 1 (SR-B1), which has been reported to help facilitate ACE2-dependent coronavirus attachment *in vitro*, reminiscent of hepatitis C virus infection. In addition, treatments targeting SR-B1 decrease the lipoprotein-mediated increment of SARS-CoV-2 infection (Wei et al., 2020).

In the second possible mechanism of liver injury, hypoxia may occur in hepatocytes following severe lung damage with SARS-CoV-2 infection, leading to an increased expression of ACE2 receptors and Hypoxia-Inducible Factors (HIFs). HIFs are one of the factors involved in hepatocyte metabolism whose increase can result in hepatic steatosis and liver injury (Li et al., 2021).

The third possible mechanism can be Drug Induced Liver Injury (DILI). Many drugs are currently used to treat patients with COVID-19, some of which may have side effects on the liver, leading to hepatocyte toxicity (Li et al., 2021). As an instance, Cai et al. disclosed that the use of lopinavir/ritonavir compounds significantly caused liver damage (Cai et al., 2020), suggesting the need for paying more attention to this drug.

Cytokine storm may be the next possible mechanism of liver injury (Alqahtani and Schattenberg 2020; Ahmed and Ahmed 2021). Cytokine storm involves the initiation of an immune response cascade due to a severe inflammatory response after COVID-19 infection. Elevated inflammatory markers such as C-Reactive Protein (CRP), Lactate Dehydrogenase (LDH), D-dimer, IL-6, IL-2, and serum ferritin lead to cytokine storms in patients with severe COVID-19, which can be followed by the sudden deterioration of the condition towards multi-organ failure and severe liver injury.

Another suggested pathway is aggravated liver damage in patients with NAFLD. Monocyte Chemotactic Protein-1 (MCP-1), also called C-C chemokine motif ligand 2 (CCL-2), was enhanced after SARS-CoV-2 infection, which aggravated steatohepatitis (Xie et al., 2016). IL-6, as the most important cytokine in the cytokine storm, also enhanced significantly in individuals with fatty liver, which could activate the innate immune response in the liver and promote the progression of liver failure (Portincasa et al., 2020b). Overall, studies have demonstrated that the upregulation of MCP-1 in COVID-19 might aggravate the progression from NAFLD to nonalcoholic steatohepatitis (Gao and Tsukamoto 2016). Hence, COVID-19 might accelerate the progression of MAFLD (Portincasa et al., 2020a; Prins and Olinga 2020). In patients suffering from severe COVID-19, a reduction was observed in visceral and peripheral blood flow, which led to hypoxia in hepatocytes (Dunn et al., 1973). Then, HIFs could further worsen MAFLD (Chen et al., 2019; Gonzalez et al., 2019). Considering the close association between COVID-19 and MAFLD, patient management is necessary during the pandemic. Lifestyle modifications such as weight loss and nutritional training may mitigate the chance and severity of COVID-19 infection and decelerate the progress of liver damage. Therefore, timely and standard diagnosis and treatment for patients with MAFLD and COVID-19 should be taken into account.

## Conclusion

Based on the recent studies, the effects of SARS-COV-2 are not limited to the respiratory system, and the disease may involve a wide range of organs. In this regard, ACE2 receptors play a critical role. The coronavirus can affect the organs involved in metabolic disorders including pancreas, liver, kidney, and gastrointestinal tract through these receptors. Subsequent to the blocking of ACE2 receptors by SARS-COV-2, a cascade of molecular pathways occurs that possibly leads to metabolic instability and the metabolic disturbance syndrome in patients as well as in recovered individuals (Chen Juan et al., 2020; Zhang et al., 2021). Therefore, patient management in terms of preventing the involvement of these organs by disrupting the pathogenesis of the virus can prevent further complications.
